# Experiences and perspectives of peer support among young adults with epilepsy

**DOI:** 10.1016/j.ebr.2023.100590

**Published:** 2023-02-01

**Authors:** Rachel Batchelor, Michelle D. Taylor

**Affiliations:** a Department of Psychology, Royal Holloway, University of London, Surrey, UK; b Health Psychology Research Limited (HPR Ltd.), 188 High Street, Egham, Surrey, UK

**Keywords:** Epilepsy, Peer support, Young adults, Long-term conditions, Mental health, YAWE, young adults with epilepsy

## Abstract

Young adulthood is a critical developmental period which having epilepsy tends to complicate, suggesting support could be useful. This study aimed to examine the experiences and perspectives of peer support among young adults with epilepsy (YAWE). An online survey was completed by 144 YAWE. Most YAWE reported not having access to peer support but perceived several potential benefits which could support their mental health including mutual understanding, fostering friendships and reducing loneliness, and promoting self-management and coping strategies. Reported barriers to accessing peer support included practical barriers such as travel and time constraints, emotional barriers (e.g., anxiety), and peers not feeling confident or skilled enough to support others. The need for epilepsy peer support groups specifically designed for young adults, university students, and individuals with co-occurring conditions such as autism and attention deficit hyperactivity disorder were also recognised as well as online delivery to address some of the identified barriers. Appropriate training for facilitators and group moderation were noted as important. Overall, these preliminary findings support the acceptability of peer support among YAWE, indicating peer support could be a worthy focus of policy and care pathway development. However, future research is needed to evaluate the effectiveness of peer support for epilepsy populations.

## Highlights

• Peer support groups specifically for young adults are desired.
• Several perceived mental health benefits of peer support are identified.
• Appropriate training for facilitators and moderation are noted as important.
• Research evaluating peer support interventions for people with epilepsy is needed.

## Introduction

Epilepsy is one of the most common long-term health conditions (LTCs), affecting over 70 million people worldwide [1]. Living with epilepsy can pose a multitude of challenges. For example, people with epilepsy (PWE) may experience stigma, discrimination, and poor employment rates [2], [3]. Additionally, PWE are at greater risk of experiencing mental health difficulties than healthy controls and people with other LTCs such as asthma and diabetes [4], [5], [6]. Risks of suicidality are also high among PWE [7], with a recent meta-analysis reporting a pooled prevalence for suicide ideation of 23.2% and suicide attempts of 7.4% in PWE [8].

Young adulthood, a critical developmental period typically characterised by increases in independence and responsibility, can be complicated by having epilepsy [9]. For example, driving is not always possible for PWE, which can impact social autonomy and career prospects [9]. In a recent systematic review, significantly lower completion rates of milestones such as higher education, leaving the parental home, and obtaining employment were reported in young adults with epilepsy (YAWE) compared with young adults with other LTCs and healthy controls [10]. Research has also shown declines in self-esteem and sense of coherence during young adulthood for YAWE [11] as well as poor mental health and quality of life outcomes [12], [13]. Despite the difficulties faced by PWE, less than half report receiving psychosocial information surrounding living with epilepsy [14] and there has been little research on the availability and effectiveness of support for YAWE.

Social support from family, friends, and significant others has been shown to predict better mental health outcomes in PWE [12], [15], [16]. Peer support is distinct from, but complementary to, other forms of support, involving people using their lived experiences to help others in similar circumstances [17], [18]. Peer support can provide a uniquely reciprocal space for people to exchange perspectives and concerns, share experiential knowledge, help find meaning, reduce isolation, and create companionship [17], [19], [20], [21].

In the context of LTCs, additional benefits of peer support have been reported such as improvements in attitudes towards illness, illness-associated identities, quality of life, illness knowledge, self-management, and retention in care [20], [21], [22], [23]. Among PWE, optimising self-management (e.g., identifying triggers, making lifestyle changes, and taking medications as prescribed) is important for improving health outcomes and reducing risks [24], [25]. Additionally, a recent scoping review on peer support and social network groups among PWE supported both emotional and instrumental functions of peer support [26]. Peer support has also been found to be a key motivating factor in participating in group intervention programs for PWE [27]. Although peer support has been shown to have benefits for the mental health of people with LTCs [28], [29], this has not previously been studied specifically in YAWE.

Previous literature indicates that peer support can have positive impacts for individuals with LTCs. Identifying experiences of peer support and perspectives of the potential impact of peer support among YAWE could inform intervention and support practices. The present study aimed to explore the experiences and perspectives of peer support among YAWE.

## Methods

### Study design

Descriptive and qualitative data from a wider cross-sectional mixed methods study on YAWE and mental health [12] was used to examine the experiences and perspectives of peer support among YAWE. A qualitative approach was incorporated to gain more personal insight.

### Participants

Participants included 144 YAWE, with a mean age of 21.60 years (*SD* = 2.20). Recruitment occurred via the UK charity ‘Epilepsy Action’ and the online community ‘Epilepsy Positivity’, primarily through social media. Eligibility was determined by the following inclusion criteria: (i) aged 18–25-years old, (ii) diagnosis of epilepsy, and (iii) living in the UK.

Sociodemographic and epilepsy-related information was gathered (Table 1). Additional epilepsy-related and clinical information for the participants have been reported elsewhere [12].

**Table 1:** Sociodemographic and epilepsy-related information for young adults with epilepsy sample (N = 144).

Sociodemographic/epilepsy-related characteristic	*n* (%)
Gender	
Female	88 (61.1%)
Male	56 (38.9%)
Ethnicity	
White British	127 (88.2%)
Other White background	9 (6.3%)
Mixed/multi-ethnic	4 (2.8%)
Asian/British Asian	2 (1.4%)
Black/African/Caribbean/Black British	2 (1.4%)
Marital status	
Single	88 (61.1%)
In a relationship	48 (33.3%)
Married/engaged	8 (5.6%)
Current occupation	
Full-time employment	55 (38.2%)
Full-time university student	41 (28.5%)
Part-time employment	27 (18.8%)
Unemployed and not in education	12 (8.3%)
Part-time university student	9 (6.3%)
Seizure type(s)	
Generalised	61 (42.4%)
Both focal and generalised	51 (35.4%)
Focal	32 (22.2%)
Time since most recent seizure	
< 30 days	84 (58.3%)
≥ 30 days	60 (41.7%)

### Ethical approval

The Research Ethics Committee at Royal Holloway, University of London, granted approval for the study. All participants were given the contact details of the researchers to enable them to ask questions about the study before deciding to take part. All participants provided informed consent before participating. Participants were debriefed and signposted to relevant epilepsy and mental health resources upon completion of the survey. To protect confidentiality, all quotes have been anonymised.

### Survey

Participants completed an online questionnaire using the survey software ‘Qualtrics’ (https://www.qualtrics.com) (online recruitment was used to increase reach). Further details on the survey are reported elsewhere [12].

In terms of peer support, participants were asked if they currently had access to peer support (‘yes’ or ‘no’). If they answered ‘yes’, they were asked to select the type(s) of peer support they had access to (e.g., ‘one-to-one support’, ‘peer support groups’, ‘online support platforms’, ‘befrienders’), with an ‘other (please specify)’ option, and select the function of this peer support (‘assistance in daily self-management’, ‘social and emotional support to encourage self-management and coping with negative emotions’, ‘provided links to clinical care and community resources’, and ‘provided ongoing support’), based on the four key functions of peer support for individuals with LTCs identified by Fisher and colleagues [30], [31]. Two additional options, ‘other (please specify)’ and ‘peer support has not been helpful to me’, were also included.

Participants who answered ‘no’ to currently having access to peer support were asked if they would access peer support if it was available (‘yes’, ‘no’, or ‘not sure’) and to select what the perceived benefits would be, using the above four functions of peer support identified by Fisher and colleagues [30], [31] as well as ‘other (please specify)’ and ‘I don’t think peer support would have any benefits’. These participants were also asked to select reasons which may stop them accessing peer support (e.g., ‘time’, ‘stigma’, ‘confidentiality and trust concerns’) along with an ‘other (please specify)’ option.

All participants were asked if they were accessing the level of peer support that they wished to (‘yes’, ‘no’, or ‘not sure’). This question was followed by an open textbox where participants were asked to suggest the types of peer support that they would like to be available and have access to. Finally, all participants were asked if they thought that peer support could be beneficial for their mental health (‘yes’, ‘no’, or ‘not sure’), followed by an open text box where participants could provide further details to explain their answer.

### Workflow

Following participatory research methodologies [32], YAWE played a fundamental role in the research process. Through the ‘Epilepsy Action Research Volunteer Network’ [33], a group of YAWE reviewed the project proposal and shaped the survey draft. YAWE also provided feedback regarding the survey’s relevance, navigation, language, and questions. Based on their feedback, edits were made to ensure questions were worded clearly and sensitively. All patient reviewers indicated in open-text feedback that the research was meaningful to the lives of YAWE.

The survey took approximately 30 minutes to complete and formed part of a broader project studying YAWE and mental health difficulties [12]. Participants were given the option to enter into a prize draw to win a £25 Amazon voucher or £25 to donate to an epilepsy charity of their choice. Data collection occurred between February 2019 and April 2019.

To protect confidentiality, all qualitative data was anonymised. Content analysis was used to explore the types of peer support the participants would like to be available and have access to, and the perspectives of peer support in relation to mental health. The conventional content analysis approach is inductive in nature, aiming to identify categories that ‘flow from the data’ [34]. This was deemed the most appropriate form of qualitative analysis given the large number of responses gathered and the requirement to identify the responses with the highest relevance. The researchers, with a mix of clinical and lived experience expertise, independently coded and analysed the data. This helped to reduce researcher bias and increase the reflexivity and rigour of the study. The researchers independently identified potential codes before meeting to discuss and define the broad categories and subcategories. Responses were then independently re-coded into these categories, disagreements were discussed, and final coding agreed. In accordance with the guidelines for agreement by Landis and Koch [35], inter-rater reliability was assessed using Cohen’s kappa coefficients.

## Results

### Access to peer support

Twenty-six (18.1.%) out of 144 YAWE reported having access to peer support. The types of peer support were: ‘spaces to hear the experiences of others’ (*n* = 12, 8.3%), ‘online support platforms’ (*n* = 11, 7.6%), ‘relaxation and mindfulness’ (*n* = 9, 6.3%), ‘peer support groups’ (*n* = 7, 4.9%), ‘linking with wider community activities’ (*n* = 4, 2.8%), ‘befrienders’ (*n* = 2, 1.4%), ‘group therapy’ (*n* = 1, 0.7%), and ‘community club for disabled young people’ (*n* = 1, 0.7%).

The majority (*n* = 71, 60.2%) of the 118 YAWE without access to peer support reported that they would access peer support if it was available to them, 36 (30.5%) were not sure, and 11 (9.3%) would not.

### Reported actual and perceived benefits of peer support

The actual benefits of peer support selected by the 26 YAWE who reported having access to peer support and the perceived benefits selected by the 118 YAWE without access to peer support are displayed in Table 2. ‘Social and emotional support to encourage self-management and coping with negative emotions’ was the most selected actual and perceived benefit, followed by providing ‘ongoing support’. There was some discrepancy between actual and perceived benefits, with a higher percentage of YAWE selecting each benefit in the perceived than actual group.

**Table 2:** Actual and perceived benefits of peer support among young adults with epilepsy (N = 144).

Benefit	Actual (*n* = 26) *n* (%)	Perceived (*n* = 118) *n* (%)
Social and emotional support to encourage self-management and coping with negative emotions	20 (76.9%)	98 (83.1%)
Providing ongoing support	17 (65.4%)	85 (72.0%)
Assistance in daily self-management	10 (38.5%)	66 (55.9%)
Providing links to clinical care and community resources	8 (30.8%)	51 (43.2%)
No benefits	2 (7.7%)	4 (3.4%)

Note: Participants could select more than one benefit.

### Barriers to accessing peer support

Among YAWE not reporting access to peer support the most common perceived barriers included ‘travel constraints’ and ‘emotional concerns (e.g., anxiety)’. The full list of selected barriers is reported in Table 3. Those who selected ‘other’ provided the following written responses: “peers not understanding my needs and making adjustments (I’m autistic)”, “possible bullying”, “lack of motivation”, and “being in denial about epilepsy”.

**Table 3:** Perceived barriers to accessing peer support among young adults with epilepsy without reported access to peer support (n = 118).

Barrier	*n* (%)
Travel constraints	60 (50.8%)
Emotional concerns (e.g., anxiety)	53 (44.9%)
Perceived lack of confidence and skills to offer support to others	47 (39.8%)
Time constraints	42 (35.6%)
Confidentiality and trust concerns	41 (34.7%)
Worries that peer support might not meet their needs (i.e., peers not having the training that professionals have had)	38 (32.2%)
Stigma (epilepsy and mental health)	19 (16.1%)
Emotional impact of offering support to others	18 (15.3%)
Lack of interest in peer support	11 (9.3%)
Not wanting to be associated with peer support services	8 (6.8%)
Other	4 (3.39%)

Note: Participants could select more than one perceived barrier.

Despite the above barriers, of the 144 YAWE, 89 (61.8%) felt they were not accessing the level of peer support they wished to, with a further 38 (26.4%) being unsure. Only seventeen (11.8%) YAWE felt they were accessing their desired level of peer support.

### Types of peer support

**Fig. 1:**
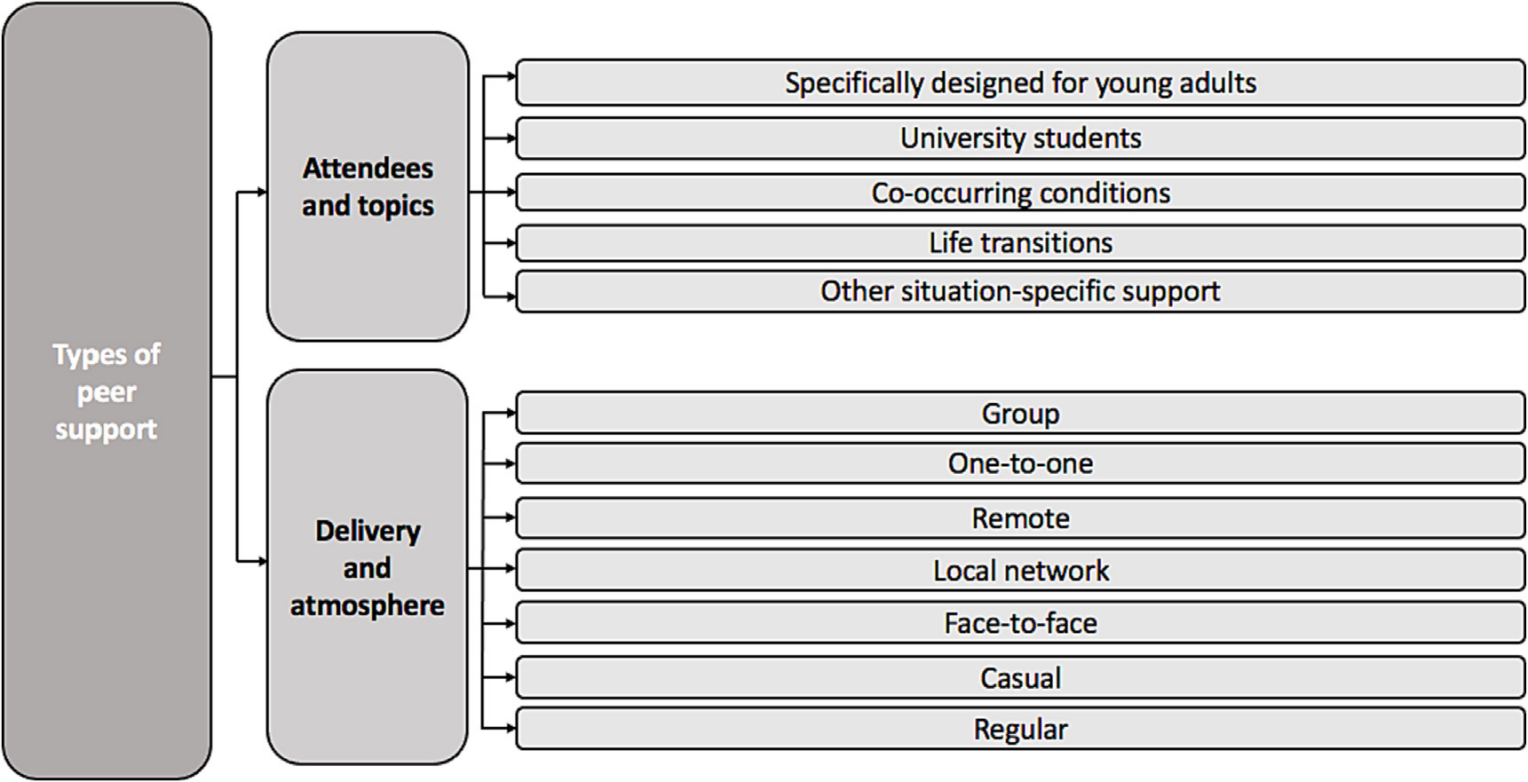
Summary of categories and subcategories of types of peer support.

Qualitative responses were provided by 103 (71.5%) YAWE, when asked to suggest the types of peer support they would like to be available and have access to. Using content analysis, responses were categorised into subcategories which were then grouped into broad categories of ‘attendees and topics’, and ‘delivery and atmosphere’, as displayed in Fig. 1. In terms of inter-rater reliability, ten subcategories had almost perfect agreement (κ = 0.82–1) and two subcategories had substantial agreement (κ = 0.63 - 0.74).

For ‘attendees and topics’, the most reported subcategory was peer support ‘specifically designed for young adults’ (*n* = 22). For example, one YAWE noted “no one understands what it’s like…going on dates, starting a new job, etc. Would be nice to speak to people my age going through the same things”. Several YAWE reflected their local peer support groups for PWE were aimed at mixed age groups: “support groups are aimed at families, children and teenagers” and “it is mainly older people who attend”.

The second most reported subcategory was peer support for ‘university students’ (*n* = 19). It was highlighted that epilepsy is relatively common: “there must be a fair number of us at university, epilepsy just isn’t spoken about!”. Peer support at university was viewed as a helpful way to encourage more awareness and inclusivity: “open up conversations, raise awareness and work together to help make campus more inclusive” for PWE.

Twelve YAWE identified the need for peer support groups to be adjusted, or specifically designed, for PWE with ‘co-occurring conditions’, namely autism and attention deficit hyperactivity disorder (ADHD): “for autistic students with epilepsy as there are unique challenges in that” and “for people with ADHD and epilepsy so I can connect with people who get me”.

Six YAWE mentioned that peer support could be helpful during ‘life transitions’. This included leaving school/education, transitioning to and from university, and transitioning to employment: “peer support to help students with epilepsy transition to university as I am so scared for next year” and “group support for young adults who have just left education and adjusting to finding or starting work”.

Ten YAWE identified a desire for ‘other situation-specific support’ and therefore “the ability to chat to people who had to make similar big decisions”. Groups specified included those for specific genders, newly/recently diagnosed, surgery, uncontrolled seizures/epilepsy, and specific types of epilepsy.

The most reported subcategory within ‘delivery and atmosphere’ was ‘group’ support (*n* = 28). Conversely, three YAWE specified wanting to access ‘one-to-one’ peer support. Fourteen YAWE expressed a desire for accessing ‘remote’ peer support. Suggestions included telephone support, online support, and social media. Several YAWE indicated that online facilitation would better suit their individual needs (for example, “online support, as I struggle with processing and social interaction”). Some participants also suggested a forum/chat to enable ongoing communication between YAWE: “some sort of moderated online forum/chat for young adults too”.

Twelve YAWE indicated a preference for a ‘local network’ of PWE in their area. A desire for building a network of PWE locally was described with the benefit of reducing travel: “more local groups as not driving can be a real struggle at times”. Six YAWE identified a preference for ‘face-to-face’ peer support. A desire for “in person connections” was described and potential settings such as clubs and youth centres were suggested. One participant recommended a blended face-to-face and online approach to peer support for YAWE: “an event for young PWE to become friends and then friendships can continue online”.

Four YAWE specified that they would prefer ‘casual’ peer support such as “doing activities together, something chilled” and “casually learning coping strategies and building independence together”. Two YAWE specified a preference for access to ‘regular’ peer support, with one suggesting “a general group meeting maybe once a week to chat”.

### Peer support and mental health

**Fig. 2:**
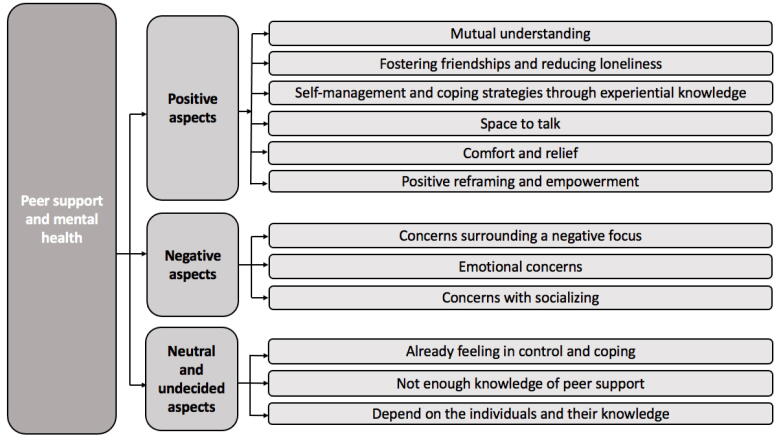
Summary of categories and subcategories of peer support and mental health.

When asked if peer support could be beneficial for their mental health, 107 (74.3%) YAWE answered ‘yes’, 30 (20.8%) answered ‘not sure’, and seven (4.9%) answered ‘no’. Qualitative responses were provided by 123 (85.4%) YAWE. Using content analysis, responses were categorised into subcategories which were then grouped into broad categories of ‘positive aspects’, ‘negative aspects’, and ‘neutral and undecided aspects’, as displayed in Fig. 2. In terms of inter-rater reliability, nine subcategories had almost perfect agreement (κ = 0.86–1) and three subcategories had substantial agreement (κ = 0.68–0.79).

The most reported subcategory within ‘positive aspects’ was ‘mutual understanding’ (*n* = 62). YAWE reported that they would value connecting with others who understand the unique challenges of living with epilepsy: “no one understands what I go through…having someone who gets it is so important” and “sharing experiences with others that know what it’s like”.

The second most reported subcategory in ‘positive aspects’ was ‘fostering friendships and reducing loneliness’ (*n* = 35). YAWE reflected that having epilepsy could feel lonely, alienating, and isolating. For example, “feeling lonely is the biggest obstacle I face daily” and “epilepsy is often misunderstood and isolating”. Participants also reflected that being able to navigate life with others with epilepsy could foster a sense of solidarity, belonging, and community as well as friendships. For example, “we could lift each other up and gain friendships”.

The third most reported subcategory in ‘positive aspects’ was ‘self-management and coping strategies through experiential knowledge’ (*n* = 23). Participants commented on being able to “learn through sharing coping strategies and hearing how other people have got through their epilepsy struggles” to “gain more independence”.

Twenty YAWE noted that peer support could facilitate a ‘space to talk’ in a safe and non-judgemental environment (*n* = 20). For example, “a space to share experiences with less risk of being judged”. Some YAWE reflected that not having a space to talk could increase stress, potentially triggering seizures: “keeping your emotions in will only make you feel more stressed, tired, angry, and could cause more seizures”.

Eighteen YAWE described feelings of ‘comfort and relief’ could arise from accessing peer support. For example, “it is more comforting talking to someone who actually knows what you're going through rather than someone who thinks they do” and “getting emotions, thoughts, and feelings off your chest can be incredibly relieving”. One participant expressed that receiving peer support could also help them to feel more comfortable with having epilepsy: “if I have [peer] support, I'll be able to be more confident and comfortable with my diagnosis”.

Seventeen YAWE expressed that peer support could help with ‘positive reframing and empowerment’ through improving their outlook and focusing on their strengths. YAWE also reported that peer support could help to reduce self-stigma and foster feelings of self-worth, hope and motivation: “help me feel empowered and focus on my strengths and potential…also the hope and motivation to keep going” and “boost my feelings of self-worth”.

For ‘negative aspects’ of peer support, the subcategory with the most responses (*n* = 5) was ‘concerns surrounding a negative focus’. For example, some participants noted that peer support “would attract all my attention to epilepsy and the negative effects”. ‘Emotional concerns’ surrounding peer support were also described by four YAWE such as “I worry about relying on others” and “I would feel guilty discussing difficulties as others may have it worse”. Three YAWE described ‘concerns with socialising’ in a peer support context and having difficulties with, or not wanting to, open up to others: “I struggle with social situations and communicating” and “I don’t like talking about my problems”.

For ‘neutral and undecided aspects’, the subcategory with the most responses was ‘already feeling in control and coping’ (*n* = 7). For example, “I already feel in control of my wellbeing” and “I can cope with a lot of daily activities”. The subcategory with the second most responses was ‘not enough knowledge or no experience of peer support’ (*n* = 6). Example responses included: “I don’t know enough about peer support” and “I’m unsure of what peer support is available and potential benefits”. Two participants commented that their decision to access peer support would be ‘dependent on the individual and their knowledge’. For example, “it would vary from person-to-person and depend on the knowledge of the peers”.

## Discussion

This study explored the experiences and perspectives of peer support among YAWE. For those who had access to peer support this included spaces to hear the experiences of others, online support platforms, relaxation and mindfulness, and support groups. YAWE who had experienced peer support reported several benefits which have been identified in previous literature among people with other LTCs [30], [31], especially social and emotional support to encourage self-management and coping with negative emotions.

Over four-fifths of YAWE did not have access to peer support, yet the majority of these individuals perceived the potential benefits outlined by Fisher and colleagues [30], [31]. Furthermore, almost two-thirds reported that they would access peer support if it was available to them. Whilst it is notable that a higher percentage of YAWE selected each benefit in the perceived than actual group, the current findings indicate motivation for peer support among YAWE. Similar to previous research, barriers to accessing peer support included practical barriers such as travel and time constraints, emotional barriers (e.g., anxiety), worries about stigma, and peers not feeling confident or skilled enough to offer support to others [26].

Qualitative responses offered insight into the types of peer support desired by YAWE. This included peer support groups specifically designed for YAWE, as opposed to broader age groups. Young adulthood is a unique life stage [9] and previous research has shown that peer support with more similar peers (e.g., similar age group), is more likely to result in understanding and mutual help [36]. Peer support for YAWE at university was also viewed as important. YAWE at university have been reported to have poorer mental health than employed YAWE [12]. YAWE reported epilepsy was not spoken about widely at university, perhaps a reflection of non-disclosure resulting from stigma [37]. The desire for peer support at university for YAWE may also reflect broader challenges faced by LTC populations at university [38], suggesting inclusivity at university requires improvement. Promising findings have been found from investigations of peer support for university students in general [39], yet further work is necessary to evaluate peer support for university students with epilepsy. Life transitions to and from university as well as employment were also identified as challenging times when peer support could be beneficial. Some participants noted that they would benefit from support from peers with similar characteristics (e.g., gender) and in similar situations (e.g., being recently diagnosed or considering surgery) to their own, reinforcing the usefulness of shared experiences.

It was recognised that peer support groups specifically designed for PWE with co-occurring conditions such as autism and ADHD [40] are important. Previous research on peer support for people with neurodevelopmental conditions such as autism and ADHD have reported promising findings [41], [42]. Evaluating peer support for individuals with both epilepsy and neurodevelopmental conditions may be a worthy avenue of future research.

Some participants expressed a preference for accessing peer support remotely. Previous literature has supported the acceptability, feasibility, and efficacy of psychological interventions for PWE and peer-support based interventions for other LTCs delivered online [21], [43], [44], [45]. Facilitating peer support online could address some of the practical barriers such as travel and time constraints [46] as well as enable the regularity of contact which was noted as important by some YAWE. Moreover, online delivery could make it more feasible for tailored groups to be disseminated, as desired. Social media, an avenue which is becoming increasingly used for health information and advice [47], was also suggested, warranting investigation.

A proportion of YAWE expressed a preference for face-to-face peer support to build connections in person, including a local network of YAWE to minimise travel barriers. A blended approach was suggested, whereby YAWE meet in person, perhaps through charity events, and continue strengthening those connections online. Preferences were mixed regarding one-to-one or group peer support as well as the activities involved, suggesting having a mix of peer support options available could be beneficial.

Qualitative responses indicated perceived benefits of peer support in relation to mental health such as mutual understanding, fostering friendships and reducing loneliness, self-management and coping strategies through experiential knowledge, having a space to talk, comfort and relief, and positive reframing and empowerment. These perceived benefits are in line with previous peer support research [20], [48]. Some benefits, such as mutual understanding, are arguably unique to peer support, considering the roles that social belonging and connectedness play in mental health and wellbeing [49]. Other benefits, such as having a space to talk, reflect the broader benefits of accessing psychosocial interventions [50].

Concerns about accessing peer support were also identified including issues surrounding a negative focus and exacerbating negative feelings, in line with previous research [48], [51], as well as the potential emotional impact of offering support to others. Indeed, the risk of revisiting negative emotions related to personal experiences when supporting peers, termed emotional entanglement, has been discussed in previous literature [20]. Concerns surrounding peers not being skilled enough to provide peer support was also identified as an access barrier. This supports previous research [48] and emphasises the importance of appropriate training for those facilitating peer support. Moreover, possible bullying was reported as a barrier in the current study, highlighting the need for potential professional moderators of peer support to further ensure safety.

### Clinical implications

This study was the first, to our knowledge, to explore perspectives and experiences of peer support specifically among YAWE. Whilst preliminary, the predominantly positive findings are promising, as peer support is a low resource-intensive form of support which can have therapeutic benefits [52]. Peer support could therefore be a worthy focus of policy development to improve care pathways for YAWE. The current study suggests peer support could be helpful for YAWE, especially when specifically for young adults and tailored to the needs of those attending. This seems particularly important, given the unique transitional period faced by this population which can be complicated by having epilepsy [9], [10], [11].

To further understand the effectiveness of peer support on outcomes such as mental health and self-management in epilepsy populations, longitudinal intervention trials are needed. Given the variability of the types of peer support that YAWE would like to access, each warrant further research to determine the acceptability, feasibility, and effectiveness of different models (i.e., types) of peer support, as demonstrated in other LTCs such as diabetes [52]. This may include peer support within group therapies for YAWE. Future research determining the components and content, such as coping skills [12], of effective peer support for YAWE is also warranted, to optimise benefits and facilitate the provision of appropriate training, moderation and guidance. Involving YAWE in the design process of such interventions may help with promoting acceptability [53] as well as ensure that the content remains relevant.

### Limitations and future directions

Whilst online recruitment enabled UK-wide participation, most participants were female and White British. This may have impacted the findings, as accessing support tends to be more challenging for males and minority groups [54], [55]. Furthermore, given the online recruitment, participation may not have been possible for YAWE without access to appropriate devices or internet connectivity. Consequently, perspectives on the types of desired peer support might already be skewed towards online modalities, highlighting the need for future research to utilise additional avenues of recruitment such as epilepsy clinics. The sample of YAWE who had received peer support and could therefore reflect on their actual experiences of it was also small. Therefore, future research should seek larger and more diverse samples to increase the generalisability of findings. Whilst the focus of this particular study was young adults, exploring peer support in other age groups of PWE may also be beneficial to inform care for wider epilepsy populations. Moreover, utilizing focus groups and interviews in future studies could explore the perceptions of peer support of PWE in more depth.

Information regarding the provision of professional support (e.g., epilepsy specialist nurses providing epilepsy management advice) was not gathered in the survey. Such support has been shown to impact PWE with their mental health and wellbeing [56], thus potentially influencing the perspectives and findings of the current study. Furthermore, the present study retrospectively gathered experiences and assumed self-reported intentional behaviour was a valid indicator of actual behaviour. Previous literature suggests that intentional behaviours for accessing support services do not always translate in the actual behaviour [57], [58], termed the intention-behaviour gap. Thus, whilst YAWE may view the concept of peer support positively, the rates of access if available remain unknown.

## Conclusion

The present study contributes to understanding the experiences and perspectives of peer support for YAWE. Most YAWE were not currently accessing the level of peer support they wished to. Several benefits unique to peer support were reported including mutual understanding, fostering friendships and reducing loneliness, and promoting self-management and coping strategies through experiential knowledge, indicating peer support could be a promising method for fostering self-management and mental health among YAWE. Whilst future research is necessary for facilitating and assessing the effectiveness of peer support interventions among epilepsy populations, the preliminary findings from the present study indicate that peer support could be a worthy focus of policy development to improve care pathways for YAWE.

## Declaration of Competing Interest

The authors declare that they have no known competing financial interests or personal relationships that could have appeared to influence the work reported in this paper.
